# Current and Emerging Targets in Immunotherapy for Osteosarcoma

**DOI:** 10.1155/2019/7035045

**Published:** 2019-01-01

**Authors:** Shinji Miwa, Toshiharu Shirai, Norio Yamamoto, Katsuhiro Hayashi, Akihiko Takeuchi, Kentaro Igarashi, Hiroyuki Tsuchiya

**Affiliations:** ^1^Department of Orthopaedic Surgery, Kanazawa University School of Medicine, Kanazawa 920-8640, Japan; ^2^Department of Orthopaedics, Graduate School of Medical Science, Kyoto Prefectural University of Medicine, Kyoto 602-8566, Japan

## Abstract

Osteosarcoma is the most common primary malignancy of bone. Although outcomes of patients with osteosarcoma have improved since the introduction of chemotherapy, outcomes of metastatic or unresectable osteosarcomas are still unsatisfactory. To improve osteosarcoma outcomes, the development of novel systemic therapies for osteosarcoma is needed. Since the 1880s, various immunotherapies have been utilized in patients with osteosarcoma and some patients have shown response to the treatment. Based on recent studies about the role of the immune system in malignancies, immunotherapies including immune modulators such as interleukin-2 and muramyl tripeptide, dendritic cells, immune checkpoint inhibitors, and engineered T cells have been utilized in patients with malignancies. Although there are limited reports of immunotherapies for osteosarcoma, immunotherapy is thought to be a promising treatment option for treating osteosarcomas. In this review, an overview of various immunotherapies for osteosarcoma is provided and their potential as adjuvant therapies is discussed.

## 1. Introduction

Osteosarcoma, the most common primary malignancy of bone, is thought to originate from mesenchymal stem cells [1]. Osteosarcoma commonly metastasizes to the lung (more than 85%) and the bone [2]. Before the introduction of chemotherapy, the outcome of patients with osteosarcoma was poor, and the survival rate of patients with osteosarcoma was less than 20% before the 1970s. The establishment of surgical resection with adequate surgical margins and neoadjuvant chemotherapy using methotrexate, doxorubicin, cisplatin, and ifosfamide increased the survival rate up to 60-70% [3]. On the other hand, outcomes of patients with recurrent, metastatic, or unresectable osteosarcomas are still unsatisfactory. The long-term survival rate for patients with localized osteosarcoma is about 65%, whereas it is less than 20% for patients with metastatic osteosarcomas [2, 4, 5]. However, no significant improvements have been seen over the last three decades, and recurrent or metastatic osteosarcomas are usually resistant to current standard treatment. Although there are increasing systemic treatment options for advanced sarcoma including pazopanib, trabectedin, and eribulin [6], the efficacy of the agents for treating patients with osteosarcoma remains unclear. Therefore, novel therapeutic approaches for advanced sarcomas have been sought to improve the treatment of osteosarcomas.

This article reviews immune surveillance for malignancy, the history of immunotherapy, and recent basic and clinical research about immunotherapy for treating osteosarcoma. Furthermore, we discuss the future perspectives of immunotherapy for osteosarcoma treatment.

## 2. Role of the Immune System and Advancement in Immunotherap

**Figure 1:**
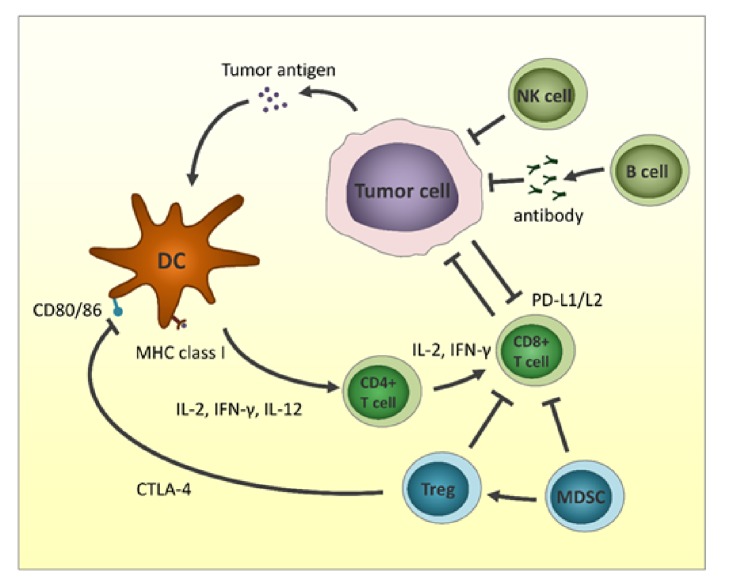
Interactions between tumor cells and microenvironment. Antitumor immune system includes dendritic cells (DCs), CD4^+^ T cells, CD8^+^T cells, natural killer (NK) cells, and tumor-suppressing killer B cells. Tumor cells escape immune surveillance by expression of immune checkpoint proteins, regulatory T (Treg) cells, and myeloid-derived suppressor cells (MDSCs).

The immune system, a complex organization of immune cells and mediators, collaborates with other accessory cells to protect against various pathogens such as viruses and bacteria. The innate immune system consists of dendritic cells (DCs), macrophages, natural killer (NK) cells, neutrophils, basophils, and eosinophils. Innate immune cells are the initial defense against foreign antigens (Figure 1). Macrophages and mast cells initiate the inflammatory response by releasing cytokines to interact with other immune cells. DCs, strong antigen-presenting cells, play a role in taking foreign antigens and presenting them for recognition by adaptive immune cells. The adoptive immune cells consist of B lymphocytes, CD4-positive T helper lymphocytes, and CD8-positive cytotoxic T lymphocytes. These cells require direct activation by antigen presentation from antigen-presenting cells. Antigen-specific T lymphocytes and B lymphocytes are generated by presentation and activation. Furthermore, the innate and adaptive cells eliminate pathogens and remove damaged cells [7, 8]. Immunosurveillance for cancer requires the recognition of tumor-specific antigens, including the products of mutated genes, overexpressed normal genes, or genes encoding viral proteins. In normal conditions, innate immune cells and adaptive immune cells detect tumor cells and eliminate them by activating NK cells, secreting interferons (IFNs), and subsequently activating DCs. However, some tumor cells can escape and survive this immune system attack by various mechanisms including loss of tumor antigens; downregulating the major histocompatibility complex (MHC) from the surface; altering the tumor microenvironment by recruiting regulatory T cells, myeloid-derived suppressor cells, and tumor-associated macrophages; upregulating inhibitory receptors on T cells, or upregulating inhibitory ligands on tumor cells [9–11].

### 2.1. Immune Modulator

**Figure 2:**
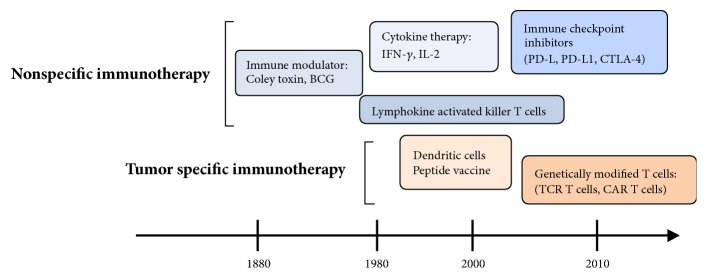
Development of immunotherapy for malignancies.

The stimulation of antitumor immune responses by bacterial infection or vaccination has been reported for over 100 years (Figure 2). In 1891, Coley reported that inducing erysipelas and stimulating immune reaction by injecting heat-inactivated* Streptococcus pyogenes* and* Serratia marcescens* (Coley's toxin) achieved complete response in approximately 10% of patients with bone and soft tissue sarcomas [12]. In recent studies on the relationship between surgical site infection and survival in dogs with osteosarcoma, infection had a positive influence on survival [13, 14]. Furthermore, Jeys reported that the 10-year survival for osteosarcoma patients with infection was 85% compared to 63% in patients without infection [15]. These reports of improved survival upon infection suggest that bacteria have the ability to activate antitumor immune responses. Larsson investigated the effects of immunotherapy using irradiated tumor cells and the Bacillus Calmette-Guerin (BCG) vaccine in a mouse model of osteosarcoma [16]. In the study, injecting irradiated tumor cells significantly prevented tumor incidence after the injection of living tumor cells, whereas BCG injection had no significant effect on osteosarcoma. Eilber reported the effect of immunotherapy consisting of BCG and an allogenic tumor cell vaccine [17]. In the study, three of 17 patients (18%) treated with BCG and tumor vaccine remained alive and disease-free, whereas 0 of 12 patients without treatment were disease-free. Karbach reported that the bacterial vaccine increased levels of immunoregulatory cytokines including interleukin (IL)-6, tumor necrosis factor (TNF)-*α*, IFN-*γ*, and IL1-*β*, which may be involved in inducing tumor regression [18]. Based on these studies, it is thought that treatment with a bacterial vaccine and inactivated tumor cells can induce antitumor immune responses.

### 2.2. Macrophage Activator

Muramyl tripeptide (MTP), a synthetic analog of a component of bacterial cell walls, has been developed as a nonspecific immune modulator [19]. MTP targets and activates macrophages. Kleinerman reported that treatment with liposome-encapsulated muramyl tripeptide phosphatidylethanolamine (L-MTP-PE) increased levels of TNF-*α* and IL-6 [20]. The liposomes encapsulating MTP-PE can deliver the agent selectively to monocytes and macrophages. After the delivery of MTP-PE to monocytes and macrophages, these cells become activated and tumoricidal [21, 22]. Chemotherapy did not interfere with L-MTP-PE stimulation of macrophage cytotoxicity in preclinical studies [23]. Meyers reported that adding MTP to conventional chemotherapy improved 6-year overall survival from 70% to 78% (P = 0.03) in patients with nonmetastatic osteosarcoma [24]. Furthermore, the risk of death at 6 years was decreased by 28% for patients receiving MTP. The statistically significant improvement is thought to be one of the greatest progresses of the treatment of patients with metastatic osteosarcoma in the last 30 years. On the other hand, a randomized phase 3 trial showed that five-year event-free survival rates for 91 patients with metastatic osteosarcoma who received L-MTP-PE and those who did not were 42% and 26%, respectively [25]. Based on the studies, MTP has been approved by the European Medicine Agency for the treatment of patients with osteosarcoma. These results suggest a decreased risk of recurrence and metastasis upon treatment with bacterial components in patients with osteosarcoma.

### 2.3. Cytokine

Several cytokines have been used for immunotherapy in patients with malignancies. IFN-*α* has the ability to induce differentiation and apoptosis, as well as to inhibit proliferation and angiogenesis, and the clinical efficacy of IFN-*α* in several malignancies has been reported [26–29]. In 1977, growth inhibitory effects of IFN-*α* in human osteosarcoma cells were reported [30]. Furthermore, growth inhibitory effects of human IFN-*α* have been reported in a mouse model of human osteosarcoma [31]. The efficacy of pegylated IFN-*α*-2b was investigated in patients with osteosarcoma by an international randomized controlled trial [32]. The study patients were treated with methotrexate, doxorubicin, and cisplatin (MAP), with or without pegylated IFN-*α*-2b. In the study, the 5-year overall survival rates in patients with MAP and MAP + IFN-*α*-2b were 81% and 84%, respectively. Although the report with a short follow-up period suggests little effect of adjuvant IFN-*α*-2b, the follow-up continues to determine long-term survival.

IL-2 induces the activation of lymphocytes and their differentiation into lymphokine-activated killer (LAK) cells which can recognize and eliminate various tumor cells [33]. Although responses to IL-2 have been reported in clinical trials in patients with various malignancies, only clinical trials for renal cell carcinoma and melanoma observed efficacy of IL-2 [34]. Only a few clinical trials of IL-2 for sarcomas have been reported [35, 36]. In a clinical trial using IL-2 with or without reinfusion of LAK for patients with metastatic osteosarcoma, 3-year event-free and overall survival rates were 34% and 45%, respectively [35]. Immunotherapy using a monoclonal antibody against the tumor-associated disialoganglioside GD2, granulocyte macrophage colony-stimulating factor (GM-CSF), and IL-2 was associated with a significantly improved outcome as compared with standard treatment in patients with neuroblastoma [36]. In a study of high-dose IL-2 treatment in relapsed pediatric sarcoma, two of the four patients with osteosarcoma showed a complete response, although severe side effects were observed, including increases in white blood cells (WBC), creatinine, *γ*-glutamyltransferase, C-reactive protein, glucose, and body weight and decreases of red blood cells, platelets, protein, albumin, and cholinesterase [37]. Based on the results of past studies, IFN-*α* and IL-2 can activate antitumor immune responses, although no significant improvement in osteosarcoma treatment outcomes has been observed in the previous trials.

### 2.4. Dendritic Cell

Recently, various kinds of cellular immunotherapy for advanced sarcomas have been developed [38]. Treatment of DCs pulsed with tumor lysate significantly increased induction of cytotoxic T lymphocyte (CTL) activity and increased the serum level IFN-*γ* [39]. DCs have been used to enhance tumor-specific immune responses because DCs are major antigen-presenting cells initiating cellular immune responses* in vivo* [40]. DCs pulsed with tumor lysate and reimplantation of cryo-treated tumors induced increased serum IFN-*γ* levels, reduced pulmonary metastases, and increased numbers of CD8-positive T lymphocytes in the metastatic areas [40]. Kawano [41] reported the effect of combination treatment with tumor lysate-pulsed DCs and an anti-cytotoxic T lymphocyte antigen-4 (CTLA-4) antibody in a mouse model of osteosarcoma. In the study, treatment with either the anti-CTLA-4 antibody or tumor lysate-pulsed DCs resulted in an increased number of CD8+ T lymphocytes, inhibition of primary and metastatic lesion growth, prolonged survival, reduced number of regulatory T lymphocytes, and increased levels of serum IFN-*γ*, and the combination of these treatments enhanced the systemic immune response. Furthermore, they reported that combination treatment with tumor lysate-pulsed DCs and anti-glucocorticoid-induced tumor necrosis factor receptor (anti-GITR) antibodies enhanced the systemic immune response [42].

In our phase 1/2 clinical trial of DC-based immunotherapy, 37 patients with bone and soft tissue sarcoma were included in the study [43]. The study patients were treated with DCs stimulated by tumor lysate, TNF-*α*, and OK-432. The patients showed increased levels of IFN-*γ* and IL-12 without severe toxicity. Among the 35 patients who were assessed for clinical responses, one patient (3%) showed partial response, 6 patients (17%) showed stable disease, and 28 patients (80%) showed progression of the disease [43]. On the other hand, a phase 1 trial using a vaccine of autologous DCs matured with tumor lysate and keyhole limpet hemocyanin (KLH) in 13 patients with relapsed osteosarcoma showed no significant toxicity, although only 2 out of 12 patients exhibited induction of specific T-cell immune response to the tumor [44]. In a phase 1 study of immunotherapy using tumor lysate and KLH, 2 of 12 patients with relapsed osteosarcoma showed induction of specific T cell immune response against the tumor although no patients had a clinical response [44]. In a phase 1 study of immunotherapy using DCs pulsed with tumor lysate, 5 of 10 pediatric patients with solid tumors showed progression control and 1 patient showed tumor regression [45]. Merchant et al. reported that 62% of patients with metastatic and/or recurrent pediatric sarcomas who were treated with immunotherapy using autologous lymphocytes, tumor lysate/KLH-pulsed DC vaccinations, and/or recombinant human IL7, showed T cell responses and responders showed prolonged survival period [46]. In clinical studies of DC-based immunotherapy, no severe adverse effects have been reported. These studies indicate that the strategy of DC vaccination in relapsed osteosarcoma appears safe and resulted in an immunological response in patients with sarcoma, although it resulted in an improved clinical outcome in only some patients. To improve cellular immunotherapy for osteosarcoma, further investigations for tumor-associated antigen (TAA) as target of the treatment, combination therapy, and favorable indication are needed.

### 2.5. Peptide Vaccine

Various vaccines targeting tumor lysates, proteins, and peptides have been used in clinical trials in patients with sarcoma [47–50]. To eliminate tumor cells, tumor vaccines have been used to stimulate patients' immune systems. In treating malignancy, TAA-specific T cells are activated by antigen-presenting cells as the tumor vaccines are presented on MHC-molecules. Various vaccines have been investigated as candidates for vaccine therapy. Tsukahara et al. reported that high expression of papillomavirus binding factor was observed in osteosarcoma cell lines and tumor tissues [48]. On the other hand, Tsuda et al. reported that high levels of SART3 were detected in osteosarcoma cell lines and osteosarcoma tissues, and SART3 induced HLA-A24-restricted tumor-specific cytotoxic T lymphocytes [49]. In a phase 2 trial of four HLA-matched peptides from 31 pooled peptides in 20 patients with refractory bone and soft tissue sarcoma, 6 patients had stable disease and 14 patients exhibited disease progression, although no severe adverse effect was observed in the study [47]. In a clinical trial of vaccine therapy for synovial sarcoma using a peptide spanning SYT-SSX fusion region (B peptide) and its HLA-A*∗*2402 anchor substitute, 6 of 12 patients treated with a mixture of the peptides had stable disease during the vaccination period, although one of the study patients developed intracerebral hemorrhage after the vaccination [50].

### 2.6. Genetically Modified T Cells

The development of gene-transfer technology has enabled the genetic transduction of T cell receptor (TCR) or chimeric antigen receptor (CAR) into conventional T cells.

Genes encoding *α* and *β* chains of TCR are introduced into T cells to generate TAA-specific TCR T cells [51]. HLA-A2-restricted TCRs which recognize several antigens, including MART-1, gp100, MAGE-A3, and NY-ESO-1, have been cloned [52–55]. TCR cell therapy demonstrated favorable outcomes in patients with melanoma and synovial sarcoma [56, 57]. Eleven of 18 patients with NY-ESO-1 positive synovial sarcoma (61%) who received autologous T cells transduced with an NY-ESO-1 reactive TCR demonstrated objective clinical responses, and the five-year survival rate was 14% [56].

Chimeric antigen receptor therapy is an adoptive immunotherapy utilizing T lymphocytes engineered with chimeric antigen receptors. CAR-T cells can recognize tumor antigens in an MHC-independent fashion. Chimeric antigen receptor is composed by an extracellular antigen recognition domain, which is called the single-chain variable fragment, and an intracellular signaling domain. CAR-T cell therapy has been used for patients with leukemia and this immunotherapy has been promising for sarcoma treatment. A phase 1/2 clinical study using HER2-specific CAR-T cells in patients with recurrent/refractory sarcoma demonstrated that 4 of 17 patients had stable disease for 12 weeks to 14 months without severe toxicity [58]. On the other hand, CAR-T cell therapy is associated with several adverse effects including cytokine release syndrome and “on-target, off-tumor” toxicity, and some of the adverse effects are life-threatening. Cytokine release syndrome is caused by intensive responses to tumor cell elimination mediated by activated lymphocytes [59]. Excessive levels of cytokines including C-reactive protein, IL-6, and IFN-*γ* are observed in patients, and high levels cause clinical syndromes including hypotension, fever, and neurological changes. “On-target, off-tumor” toxicity is caused by recognition of tumor-associated antigens on the surface of normal cells, and lymphocytes subsequently attack, causing damage to normal tissue. CAR-T cells may kill normal cells with target antigen even if the expression of the target antigen is at low level. Therefore, CAR-T cell therapy can be applied for cancers with specific expression of the antigen.

Although further studies of TAAs with high specificity are needed for the use of tumor-specific immunotherapies, adoptive cell therapy using TCR or CAR targeting TAAs represents a new and promising therapeutic approach for patients with sarcomas. Randomized clinical trials are demanded to assess the efficacies and safety of adoptive cell therapies in patients with sarcomas.

### 2.7. Immune Checkpoint Inhibitors

Recent studies have focused on the association of immune checkpoints with inhibition of the tumor immune system. Although immune checkpoints are necessary for maintaining self-tolerance and for limiting immune responses to prevent autoimmune disorders, immune checkpoints can allow immune tolerance against tumors. CTLA-4 and programmed death receptor-1 (PD-1), the main inhibitory receptors expressed on T cells, have been considered as an important part in immune suppression induced by tumor cells, and these molecules are thought to be candidates as new therapeutic targets in various types of advanced malignancies [60]. Activated T cells normally express PD-1 on the surface and suppress excessive immune responses, including autoimmune reactions. PD-L1, the ligand of PD-1, is expressed in various cells including macrophages and tumor cells, and the interaction between PD-1 and PD-L1 is usually influenced by tumor tissues. Dhupkar P et al. reported that anit-PD-1 therapy redirecting M2 macrophages (immunosuppressive and tumor promoting) to M1 (anti-tumor) resulted in regression of lung metastasis in mouse model of osteosarcoma [61].

Blockade of PD-1 by anti-PD-1 nivolumab or anti-PD-L1 BMS-936559 has demonstrated objective responses and improved oncological survival in patients with lung cancer, melanoma, renal cell cancer, and ovarian cancer [62, 63]. Furthermore, ipilimumab, a monoclonal antibody (mAb) targeting CTLA-4, showed significant improvement of overall survival in patients with metastatic melanomas [64]. However, no correlation with immune checkpoints has been reported in some types of sarcoma. In sarcomas with high PD-1 expression, PD-1 inhibitors are considered to be promising, while tumor antigen-specific treatments, such as dendritic cell-based immunotherapy, can be a candidate treatment in sarcomas with a high expression of tumor-specific antigen.

Murine studies suggest activity of checkpoint inhibitors in osteosarcoma [65]. In patients with osteosarcoma, PD-1 and PD-L1 levels negatively correlate with prognosis [66]. Zheng investigated the efficacy of nivolumab in a mouse model of osteosarcoma and reported that nivolumab-treated mice had significantly fewer metastatic lung lesions, although nivolumab had no effect on primary tumor volume and growth [67]. Shen et al. reported that high expression of PD-L1 was observed in osteosarcoma patients and expression of PD-L1 was correlated with tumor-infiltrating lymphocytes (TILs) [68]. In the study, median overall survival for patients with low PD-L1 expression was 89 months compared with 28 months for patients with high PD-L1 expression. Hingorani et al. reported that increased expression of CTLA-4 in T cells and increased immunosuppressive monocytes were observed in patients with pediatric sarcoma [69]. A recent study reported that high levels of PD-1 expression on peripheral CD4+ and CD8+ T cells were observed in patients with osteosarcoma, and the expression levels of PD-1 on CD4+ T cells in patients with metastasis were significantly higher than those without metastasis [66]. Lussier et al. reported that T cells infiltrating PD-L1 antibody-resistant tumors upregulated inhibitory receptors such as CTLA-4 [65]. Furthermore, combination immunotherapy using anti-CTLA-4 and anti-PD-L1 antibodies improved overall survival in a mouse model of osteosarcoma, whereas no benefit was observed upon treatment with an anti-CTLA-4 antibody alone [65]. Based on a study of immune checkpoints in patients with osteosarcoma, PD-1, PD-L1, and CTLA-4 influence osteosarcoma progression. Therefore, immune checkpoints are promising targets for treating various malignancies including osteosarcoma. The recent studies of osteosarcoma indicate that immune checkpoint can be promising treatment for patients with osteosarcoma. It is reported that the expression of checkpoint molecules such as PD-L1 on osteosarcoma cells correlates with metastases and overall survival [70]. A multicenter, phase 2 trial of the anti-PD-1 mAb pembrolizumab demonstrated that seven of 40 patients with bone and soft tissue sarcoma (18%) and two of 40 patients (5%) had objective responses [71]; treatment-related serious adverse events occurred in 11% of patients. The study included 22 patients with osteosarcoma; one patient (5%) had a partial response, 6 patients (27%) had stable disease, and 15 patients (68%) exhibited disease progression. It is considered that immune checkpoints play some roles in sarcoma development, and immune checkpoints can be promising targets for osteosarcoma treatment. Further basic and clinical studies will determine the efficacy of immune checkpoint inhibitors.

### 2.8. Conclusions

Based on recent studies of the tumor microenvironment, mechanisms of tumor invasion and metastasis, antitumor immune system, and immune checkpoints in malignancies, significant improvements in outcome have been seen for some malignancies. Therefore, immunotherapy is an increasingly attractive treatment option in patients with osteosarcoma. Although the introduction of chemotherapy dramatically improved the outcomes of osteosarcoma treatment, no marked improvement of the treatment for osteosarcoma has been seen in the last three decades. The main reasons for the lack of development of osteosarcoma treatment include this type of cancer's rarity, heterogeneity, and the lack of discovery of a tumor-specific antigen. For successful osteosarcoma immunotherapy, elucidation of the condition of immunosurveillance, discovery of tumor-specific antigen for osteosarcoma, and collaborative multicenter studies are necessary.
